# Supplementary material for: Sorting at embryonic boundaries requires high heterotypic interfacial tension
Source: Nat Commun. 2017 Jul 31;8:157. doi: 10.1038/s41467-017-00146-x (PMC5537356; doi:10.1038/s41467-017-00146-x)
Supplement: Supplementary file 2 — Supplementary Software 1 [file 41467_2017_146_MOESM2_ESM.zip › PottsModel/SrcPottsModel/doc/utils/HSLColor.html]

HSLColor


JavaScript is disabled on your browser.


Skip navigation links


- Overview
- Package
- Class
- Use
- Tree
- Deprecated
- Index
- Help

- Prev Class
- Next Class

- Frames
- No Frames

- All Classes

- Summary:
- Nested |
- Field |
- Constr |
- Method

- Detail:
- Field |
- Constr |
- Method


utils

## Class HSLColor

- java.lang.Object
- - utils.HSLColor

- ---

    

  ```
  public class HSLColor
  extends java.lang.Object
  ```

  The HSLColor class provides methods to manipulate HSL (Hue, Saturation
  Luminance) values to create a corresponding Color object using the RGB
  ColorSpace.
  The HUE is the color, the Saturation is the purity of the color (with
  respect to grey) and Luminance is the brightness of the color (with respect
  to black and white)
  The Hue is specified as an angel between 0 - 360 degrees where red is 0,
  green is 120 and blue is 240. In between you have the colors of the rainbow.
  Saturation is specified as a percentage between 0 - 100 where 100 is fully
  saturated and 0 approaches gray. Luminance is specified as a percentage
  between 0 - 100 where 0 is black and 100 is white.
  In particular the HSL color space makes it easier change the Tone or Shade
  of a color by adjusting the luminance value.

- - ### Field Summary

    Fields

    | Modifier and Type | Field and Description |
    | --- | --- |
    | `static HSLColor` | `Black` |
  - ### Constructor Summary

    Constructors

    Constructor and Description

    `HSLColor(java.awt.Color rgb)`

    Create a HSLColor object using an RGB Color object.

    `HSLColor(float[] hsl)`

    Create a HSLColor object using an an array containing the
    individual HSL values and with a default alpha value of 1.

    `HSLColor(float[] hsl,
    float alpha)`

    Create a HSLColor object using an an array containing the
    individual HSL values.

    `HSLColor(float h,
    float s,
    float l)`

    Create a HSLColor object using individual HSL values and a default
    alpha value of 1.0.

    `HSLColor(float h,
    float s,
    float l,
    float alpha)`

    Create a HSLColor object using individual HSL values.
  - ### Method Summary

    All Methods Static Methods Instance Methods Concrete Methods

    | Modifier and Type | Method and Description |
    | --- | --- |
    | `java.awt.Color` | `adjustHue(float degrees)` Create a RGB Color object based on this HSLColor with a different Hue value. |
    | `java.awt.Color` | `adjustLuminance(float percent)` Create a RGB Color object based on this HSLColor with a different Luminance value. |
    | `java.awt.Color` | `adjustSaturation(float percent)` Create a RGB Color object based on this HSLColor with a different Saturation value. |
    | `java.awt.Color` | `adjustShade(float percent)` Create a RGB Color object based on this HSLColor with a different Shade. |
    | `java.awt.Color` | `adjustTone(float percent)` Create a RGB Color object based on this HSLColor with a different Tone. |
    | `static java.awt.Color[]` | `colorScale(java.awt.Color rgb, int bins)` Returns a color scale from light to dark. |
    | `static java.awt.Color[]` | `colorScale(java.awt.Color rgb, int bins, int minTone, int maxTone)` |
    | `static float[]` | `fromRGB(java.awt.Color color)` Convert a RGB Color to it corresponding HSL values. |
    | `float` | `getAlpha()` Get the Alpha value. |
    | `java.awt.Color` | `getComplementary()` Create a RGB Color object that is the complementary color of this HSLColor. |
    | `float[]` | `getHSL()` Get the HSL values. |
    | `float` | `getHue()` Get the Hue value. |
    | `float` | `getLuminance()` Get the Luminance value. |
    | `java.awt.Color` | `getRGB()` Get the RGB Color object represented by this HDLColor. |
    | `float` | `getSaturation()` Get the Saturation value. |
    | `static void` | `main(java.lang.String[] args)` |
    | `static java.awt.Color` | `toRGB(float[] hsl)` Convert HSL values to a RGB Color with a default alpha value of 1. |
    | `static java.awt.Color` | `toRGB(float[] hsl, float alpha)` Convert HSL values to a RGB Color. |
    | `static java.awt.Color` | `toRGB(float h, float s, float l)` Convert HSL values to a RGB Color with a default alpha value of 1. |
    | `static java.awt.Color` | `toRGB(float h, float s, float l, float alpha)` Convert HSL values to a RGB Color. |
    | `java.lang.String` | `toString()` |

    - ### Methods inherited from class java.lang.Object

      `equals, getClass, hashCode, notify, notifyAll, wait, wait, wait`

- - ### Field Detail


    - #### Black

      ```
      public static HSLColor Black
      ```
  - ### Constructor Detail


    - #### HSLColor

      ```
      public HSLColor(java.awt.Color rgb)
      ```

      Create a HSLColor object using an RGB Color object.

      Parameters:
      :   `rgb` - the RGB Color object


    - #### HSLColor

      ```
      public HSLColor(float h,
                      float s,
                      float l)
      ```

      Create a HSLColor object using individual HSL values and a default
      alpha value of 1.0.

      Parameters:
      :   `h` - is the Hue value in degrees between 0 - 360
      :   `s` - is the Saturation percentage between 0 - 100
      :   `l` - is the Lumanance percentage between 0 - 100


    - #### HSLColor

      ```
      public HSLColor(float h,
                      float s,
                      float l,
                      float alpha)
      ```

      Create a HSLColor object using individual HSL values.

      Parameters:
      :   `h` - the Hue value in degrees between 0 - 360
      :   `s` - the Saturation percentage between 0 - 100
      :   `l` - the Lumanance percentage between 0 - 100
      :   `alpha` - the alpha value between 0 - 1


    - #### HSLColor

      ```
      public HSLColor(float[] hsl)
      ```

      Create a HSLColor object using an an array containing the
      individual HSL values and with a default alpha value of 1.

      Parameters:
      :   `hsl` - array containing HSL values


    - #### HSLColor

      ```
      public HSLColor(float[] hsl,
                      float alpha)
      ```

      Create a HSLColor object using an an array containing the
      individual HSL values.

      Parameters:
      :   `hsl` - array containing HSL values
      :   `alpha` - the alpha value between 0 - 1
  - ### Method Detail


    - #### adjustHue

      ```
      public java.awt.Color adjustHue(float degrees)
      ```

      Create a RGB Color object based on this HSLColor with a different
      Hue value. The degrees specified is an absolute value.

      Parameters:
      :   `degrees` - - the Hue value between 0 - 360

      Returns:
      :   the RGB Color object


    - #### adjustLuminance

      ```
      public java.awt.Color adjustLuminance(float percent)
      ```

      Create a RGB Color object based on this HSLColor with a different
      Luminance value. The percent specified is an absolute value.

      Parameters:
      :   `percent` - - the Luminance value between 0 - 100

      Returns:
      :   the RGB Color object


    - #### adjustSaturation

      ```
      public java.awt.Color adjustSaturation(float percent)
      ```

      Create a RGB Color object based on this HSLColor with a different
      Saturation value. The percent specified is an absolute value.

      Parameters:
      :   `percent` - - the Saturation value between 0 - 100

      Returns:
      :   the RGB Color object


    - #### adjustShade

      ```
      public java.awt.Color adjustShade(float percent)
      ```

      Create a RGB Color object based on this HSLColor with a different
      Shade. Changing the shade will return a darker color. The percent
      specified is a relative value.

      Parameters:
      :   `percent` - - the value between 0 - 100

      Returns:
      :   the RGB Color object


    - #### adjustTone

      ```
      public java.awt.Color adjustTone(float percent)
      ```

      Create a RGB Color object based on this HSLColor with a different
      Tone. Changing the tone will return a lighter color. The percent
      specified is a relative value.

      Parameters:
      :   `percent` - - the value between 0 - 100

      Returns:
      :   the RGB Color object


    - #### getAlpha

      ```
      public float getAlpha()
      ```

      Get the Alpha value.

      Returns:
      :   the Alpha value.


    - #### getComplementary

      ```
      public java.awt.Color getComplementary()
      ```

      Create a RGB Color object that is the complementary color of this
      HSLColor. This is a convenience method. The complementary color is
      determined by adding 180 degrees to the Hue value.

      Returns:
      :   the RGB Color object


    - #### getHue

      ```
      public float getHue()
      ```

      Get the Hue value.

      Returns:
      :   the Hue value.


    - #### getHSL

      ```
      public float[] getHSL()
      ```

      Get the HSL values.

      Returns:
      :   the HSL values.


    - #### getLuminance

      ```
      public float getLuminance()
      ```

      Get the Luminance value.

      Returns:
      :   the Luminance value.


    - #### getRGB

      ```
      public java.awt.Color getRGB()
      ```

      Get the RGB Color object represented by this HDLColor.

      Returns:
      :   the RGB Color object.


    - #### getSaturation

      ```
      public float getSaturation()
      ```

      Get the Saturation value.

      Returns:
      :   the Saturation value.


    - #### toString

      ```
      public java.lang.String toString()
      ```

      Overrides:
      :   `toString` in class `java.lang.Object`


    - #### fromRGB

      ```
      public static float[] fromRGB(java.awt.Color color)
      ```

      Convert a RGB Color to it corresponding HSL values.

      Returns:
      :   an array containing the 3 HSL values.


    - #### toRGB

      ```
      public static java.awt.Color toRGB(float[] hsl)
      ```

      Convert HSL values to a RGB Color with a default alpha value of 1.
      H (Hue) is specified as degrees in the range 0 - 360.
      S (Saturation) is specified as a percentage in the range 1 - 100.
      L (Lumanance) is specified as a percentage in the range 1 - 100.

      Parameters:
      :   `hsl` - an array containing the 3 HSL values


    - #### toRGB

      ```
      public static java.awt.Color toRGB(float[] hsl,
                                         float alpha)
      ```

      Convert HSL values to a RGB Color.
      H (Hue) is specified as degrees in the range 0 - 360.
      S (Saturation) is specified as a percentage in the range 1 - 100.
      L (Lumanance) is specified as a percentage in the range 1 - 100.

      Parameters:
      :   `hsl` - an array containing the 3 HSL values
      :   `alpha` - the alpha value between 0 - 1


    - #### toRGB

      ```
      public static java.awt.Color toRGB(float h,
                                         float s,
                                         float l)
      ```

      Convert HSL values to a RGB Color with a default alpha value of 1.

      Parameters:
      :   `h` - Hue is specified as degrees in the range 0 - 360.
      :   `s` - Saturation is specified as a percentage in the range 1 - 100.
      :   `l` - Lumanance is specified as a percentage in the range 1 - 100.


    - #### toRGB

      ```
      public static java.awt.Color toRGB(float h,
                                         float s,
                                         float l,
                                         float alpha)
      ```

      Convert HSL values to a RGB Color.

      Parameters:
      :   `h` - Hue is specified as degrees in the range 0 - 360.
      :   `s` - Saturation is specified as a percentage in the range 1 - 100.
      :   `l` - Lumanance is specified as a percentage in the range 1 - 100.
      :   `alpha` - the alpha value between 0 - 1


    - #### colorScale

      ```
      public static java.awt.Color[] colorScale(java.awt.Color rgb,
                                                int bins)
      ```

      Returns a color scale from light to dark.

      Parameters:
      :   `rgb` -

      Returns:


    - #### colorScale

      ```
      public static java.awt.Color[] colorScale(java.awt.Color rgb,
                                                int bins,
                                                int minTone,
                                                int maxTone)
      ```


    - #### main

      ```
      public static void main(java.lang.String[] args)
      ```


Skip navigation links


- Overview
- Package
- Class
- Use
- Tree
- Deprecated
- Index
- Help

- Prev Class
- Next Class

- Frames
- No Frames

- All Classes

- Summary:
- Nested |
- Field |
- Constr |
- Method

- Detail:
- Field |
- Constr |
- Method
